# ﻿A new species of the genus *Conosiphon* Becker, 1923 and the first records of this genus for Europe (Diptera, Asilidae)

**DOI:** 10.3897/zookeys.1181.105663

**Published:** 2023-10-02

**Authors:** Reinoud van den Broek, Piluca Álvarez Fidalgo, John Smit

**Affiliations:** 1 Insecten Werkgroep KNNV-Natuurmuseum Brabant, Spoorlaan 434, 5038CH, Tilburg, Netherlands Insecten Werkgroep KNNV-Natuurmuseum Brabant Tilburg Netherlands; 2 Collection of Entomology, Museo Nacional de Ciencias Naturales (MNCN-CSIC), c/ José Gutiérrez Abascal 2, 28006, Madrid, Spain Museo Nacional de Ciencias Naturales (MNCN-CSIC) Madrid Spain; 3 European Invertebrate Survey – the Netherlands / Naturalis Biodiversity Center, PO Box 9517, 2300 RA, Leiden, Netherlands European Invertebrate Survey – the Netherlands / Naturalis Biodiversity Center Leiden Netherlands

**Keywords:** Almería, Asilinae, endemic, Iberian Peninsula, identification key, Spain

## Abstract

A new species of *Conosiphon* Becker, 1923, *Conosiphonianus* Álvarez Fidalgo & van den Broek, **sp. nov.**, is described from Spain, representing the first record of this genus for Europe. It is illustrated in high-resolution photographs and the first ecological information is provided, as well as a key to all species tentatively placed in this genus.

## ﻿Introduction

In this paper we describe a new, possibly endemic, species of the genus *Conosiphon* Becker, 1923 from Almería, part of the Mediterranean area of Spain. The Mediterranean Basin is one of 25 well known biodiversity hotspots in the World, which contain both significant reservoirs of biodiversity and/or endemic species, and are threatened due to the loss of their original pristine habitat (Brooks et al. 2002; Myers et al. 2008). Furthermore, the Mediterranean basin is by far the most important area for conservation in Europe (López-López et al. 2011). Within the Mediterranean Basin, the Iberian Peninsula is of particular interest due to its combination of Mediterranean climate, serving as a refuge for the flora of continental Europe during glaciations, and a remarkably wide variety of habitats (Wood et al. 2021). In particular, Eastern Andalusia is an exceptional focal point mainly due to its strategic position acting as a bridge for the Iberian Peninsula and North Africa at the end of the Miocene (Cueto et al. 2014).

Almería is the easternmost province of Andalusia and is located in the southeast of Spain. The most pronounced characteristic of its natural landscape is its dryness, typical of the Mediterranean Basin, which is increased due to its geographical position and due to the relief of the landscape, which prevents the humid air masses from the Atlantic from penetrating (Castillo-Requena 1989). This makes Almería unique in Europe, not only due to the high degree of biodiversity with many species adapted to extreme conditions (Castillo-Requena 1989) but also because even at the beginning of the 21^st^ century it is still frequently providing new species of invertebrates for science (Anichtchenko 2005; Castro Tovar 2014; Pagola-Carte 2017; van den Broek and Álvarez Fidalgo 2018; Carles-Tolrá 2020; Pollet et al. 2022).

The new species of *Conosiphon* described here is the first record of this genus from Europe, which is otherwise known from the Mediterranean part of Africa. Nothing has previously been reported on the ecology of this genus; therefore, we provide the first ecological information on the genus *Conosiphon*.

## ﻿Materials and methods

The terminology used to designate the morphology follows Cumming and Monty Wood (2017). The following abbreviations for institutions, collections and collectors are used: Museo Nacional de Ciencias Naturales, Madrid, Spain (**MNCN**), Naturalis Biodiversity Center, Leiden, the Netherlands (**RMNH**), Museum für Naturkunde, Berlin (**ZMHB**), National Museum of Natural History, Smithsonian Institution, Washington, DC, USA (**USNM**), Koninklijk Belgisch Instituut voor Natuurwetenschappen, Brussels (**KBIN**), private collection of Reinoud van den Broek, Tilburg, the Netherlands (**RVDBC**), private collection of Piluca Álvarez Fidalgo, Madrid, Spain (**PAFC**).

The 52 specimens of the new species studied in this work come mainly from captures carried out by Francisco Rodríguez Luque using entomological nets, and regulated by capture authorisations issued by Consejería de Medio Ambiente y Ordenación del Territorio de Almería. The holotype and 11 paratypes are stored at MNCN and the 40 remaining paratypes at RMNH, ZMHB, USNM, and in the private collections of the first two authors. Additionally, type specimens of *Conosiphonpauper* (Becker, 1907) and *Conosiphonsimilis* Becker, 1923 were studied through high resolution images provided by ZMHB.

Morphological study of all the pinned specimens was performed with an Olympus X-Tr, an Olympus VB 454, and a Leica M80 binocular microscope. The genitalia of some male specimens were first detached from the abdomen and then macerated for 24 hours in a cold solution of approximately 20% KOH; afterwards, they were dissected and prepared on glycerine in order to be photographed. High-resolution photographs of the genitalia were taken using a Nikon D810 camera equipped with a Cnscope 4X Achromatic Microscope Objective Lens with extension tube; Zerene Stacker 1.04 software was used in order to obtain fully focused images, and afterwards the Adobe Photoshop CC 2015 program was used to improve the lighting and contrast. Based on these photographs, the drawings were performed. The male holotype and one female paratype were photographed in high resolution with a Nikon D810 camera and a Nikon Nikkor 105 mm macro lens with a Kenko 36 mm extension tube and using the stacking technique of the Helicon Focus v. 7.6.4 software. The maps were created by using the software program QGIS 3.4. Field images were taken using reflex cameras (Nikon D5300 and Canon EOS 600D) fitted with macro lenses (Nikon 60 mm and Tamron 180 mm).

DNA extraction was conducted on single legs, using the NucleoMag 96 Tissue kit by Macherey-Nagel on a Thermo Scientific KingFisher Flex magnetic bead extraction robot, with a final elution volume of 150 µl. The standard COI barcoding fragment (Hebert et al. 2003) was amplified using a cocktail of primers LCO1490 and HCO2198 (Folmer et al. 1994), and LepF1 and LepR1 (Hebert et al. 2004). PCR reactions contained 18.75 µl mQ, 2.5 µl 10× PCR buffer CL, 1.0 µl 10 mM of each primer, 0.5 µl 2.5 mM dNTPs and 0.25 µl 5U Qiagen Taq, with 1.0 µl of template DNA. PCR was performed using an initial denaturation step of 180 sec at 94 °C, followed by 40 cycles of 15 sec at 94 °C, 30 sec at 50 °C and 40 sec at 72 °C, and finishing with an extension of 300 sec at 72 °C and pause at 12 °C. Bidirectional sequencing was performed at BaseClear (www.baseclear.com/). Sequences were edited manually with Sequencher v. 4.10.1 (Gene Codes Corporation). For all barcoded specimens, sequences and collection data were uploaded to the Barcode of Life Database (BOLD, www.boldsystems.org/). BOLD accession codes are provided for the specimens that produced DNA barcodes.

## ﻿Taxonomic account

### ﻿ Conosiphon

Taxon classificationAnimaliaDipteraAsilidae

Becker, 1923

F301540B-1CC3-581A-B5F7-48CCE393C14E

#### Type species.

*Dysmachuspauper* Becker, 1907 (by original designation); type locality: Algeria.

This Palearctic genus was originally proposed by Becker (1923: 36) as a subgenus of *Machimus* Loew, 1849 sensu stricto based on the combination of the long macrosetae on the mesonotum in both the male and the female (similar to that of the genus *Dysmachus* Loew, 1860, and very different from typical *Machimus*, without macrosetae on the prescutum) and the shape of the ovipositor of the female (very different from that of *Dysmachus*, with cerci partly incorporated in the ovipositor in tergite IX, and closer to that of *Machimus*, with cerci sticking out at the apex). Lehr (1967) raised it to genus status but did not give a convincing justification as he only states ‘It is essential to promote the subgenus to the rank of genus on the same grounds we had for taking *Eutolmus* out of *Dysmachus*’, which is confusing as the genus *Eutolmus* Loew, 1848 was never included in *Dysmachus*. However, considering the differences in the three taxa, in our opinion *Conosiphon* clearly deserves the status of genus.

Seven species have been assigned to the genus *Conosiphon* at some point in history but four of these are now considered to belong to other genera. Two of them were treated as such by Engel (1930), *Asilusfuscus* Macquart, 1839 and *Tolmeruscorsicus* Schiner, 1867. *Asilusfuscus* was described from the western Canary Islands where it is considered endemic (Izquierdo et al. 2001) and it is nowadays treated as a species of the genus *Tolmerus* (Hradský and Bosák 2006). This species was erroneously mentioned from southern Russia by Engel (1930). *Tolmeruscorsicus* was described from Corsica, was included in Engel 1930’s key in the subgenus Conosiphon, but was treated as *Tolmerus* in his text and it is now assigned to genus *Machimus* (Evenhuis and Pape 2023). *Machimussagittarius* (Villeneuve, 1930) from Algeria was originally described as a *Conosiphon* but Lehr (1988) assigned it to *Machimus*. The fourth species, *Minicatusmirabilis* (Lehr, 1967) from Kazakhstan and Soviet Middle Asia was also originally described as a *Conosiphon* but Lehr himself finally established the genus *Minicatus* (Lehr, 1992) for this species, without explaining the reasons. However, he does mention that the internal side of the gonocoxite has a rugose surface that forms more than 15 grooves and that the gonostylus is plate-shaped, both characters of which are very different from those of the species here presented. Therefore, we consider four species, including the one described in this paper, to belong to this genus which fits the conclusions by Lehr (1988):

- *Conosiphonalter* Becker, 1923: (originally described in the genus *Conosiphon*). Type material in the Collection Villeneuve (Becker, 1923), which is stored at KBIN. However, the collection was carefully checked by Reinoud van den Broek and Jonas Mortelmans and the specimens were not found. Type material might be lost.
- *Conosiphonianus* Álvarez Fidalgo & van den Broek sp. nov. Type material stored at MNCN, RMNH, ZMHB and USNM.
- *Conosiphonpauper* (Becker, 1907): (originally described in the genus *Dysmachus*). Type material stored at ZMHB.
- *Conosiphonsimilis* Becker, 1923: (originally described in the genus *Conosiphon*). Type material stored at ZMHB.

#### Diagnosis.

After the diagnosis based on Hull (1962), who in turn, based his diagnosis of the genus on the works by Becker (1923) and Engel (1930), *Conosiphon* can be separated from other genera of Asilinae by the following characters combined: a) facial protuberance strong and prominent, occupying most of the face, its upper margin well developed, b) dorsocentral macrosetae set all over the central area of the mesonotum, from the front to the back, and c) the paired cerci of ovipositor are partly incorporated in the ovipositor in tergite IX. In other words, external appearance of the male is similar to genus *Dysmachus*, but in the female, the cerci of ovipositor are not wedged in as in *Dysmachus*, but style-like in lateral view.

### ﻿ Conosiphon ianus

Taxon classificationAnimaliaDipteraAsilidae

Álvarez Fidalgo & van den Broek sp. nov.

525BE411-2B44-51BB-B7EB-2D8693765BF7

https://zoobank.org/4FF97738-4B10-46E4-AA90-3F2026A3FAB5

**Figure 1.**
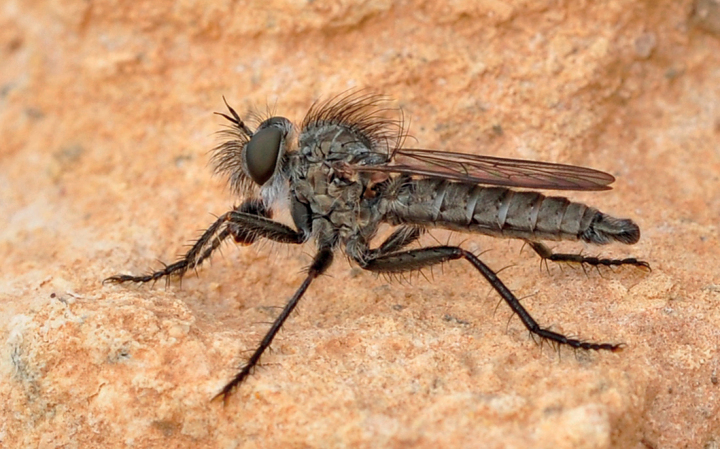
*Conosiphonianus* Álvarez Fidalgo & van den Broek, sp. nov. Male (clear-winged form) in its natural habitat, El Parador (Roquetas de Mar), Almería, Spain, 25-XII-2009. Photograph: F. Rodríguez Luque.

**Figure 2.**
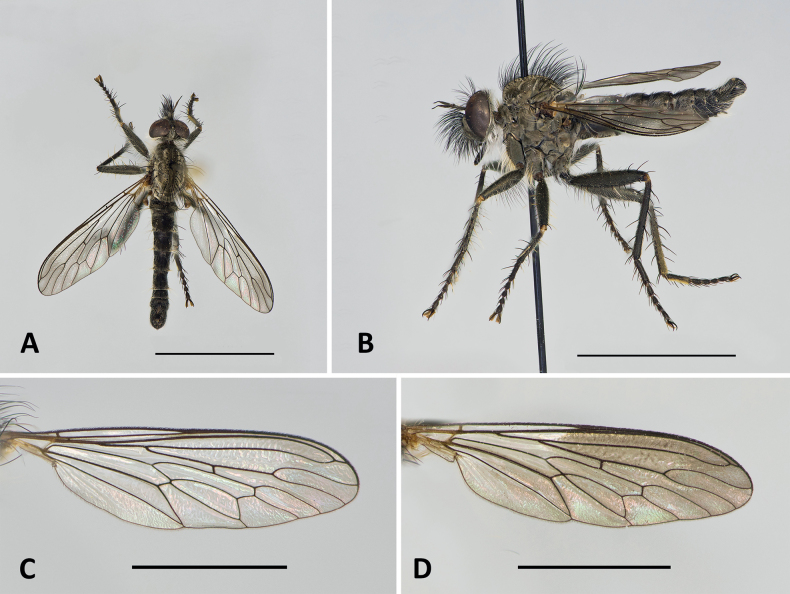
*Conosiphonianus* Álvarez Fidalgo & van den Broek, sp. nov., male holotype (MNCN_Ent 344922) **A** habitus in dorsal view **B** habitus in lateral view Male paratype of the clear-winged form (PAFC) **C** wing. Male paratype of the dark-winged form (PAFC) **D** wing. Scale bars: 5 mm (**A, B**); 3 mm (**C, D**). Photographs: PÁF.

**Figure 3.**
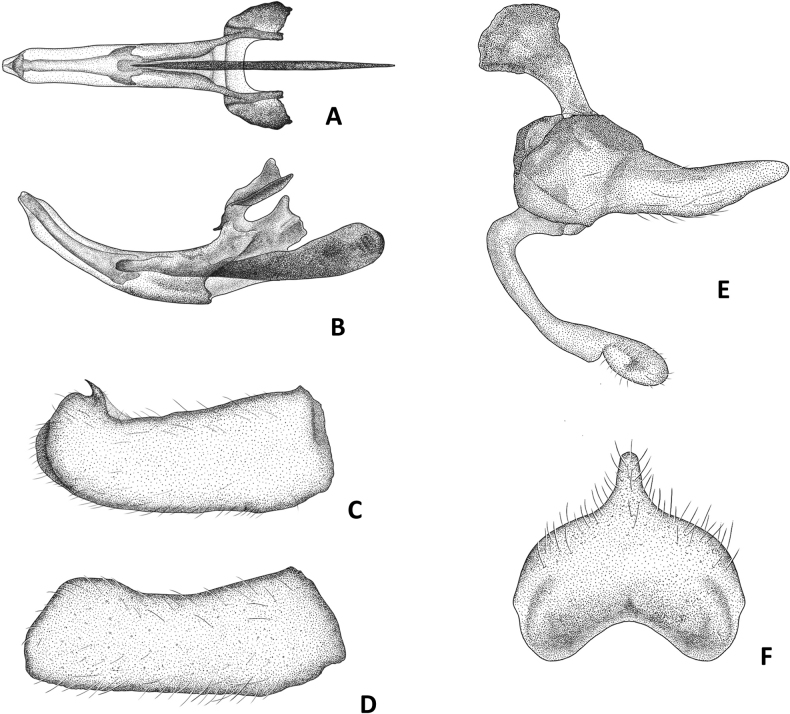
*Conosiphonianus* Álvarez Fidalgo & van den Broek, sp. nov., male terminalia **A** phallus in dorsal view **B** phallus in lateral view **C** right epandrium in dorsal view **D** right epandrium in lateral view **E** left gonocoxite and gonostylus in lateral view **F** hypandrium. Drawings: M. Trillo Camprodón.

**Figure 4.**
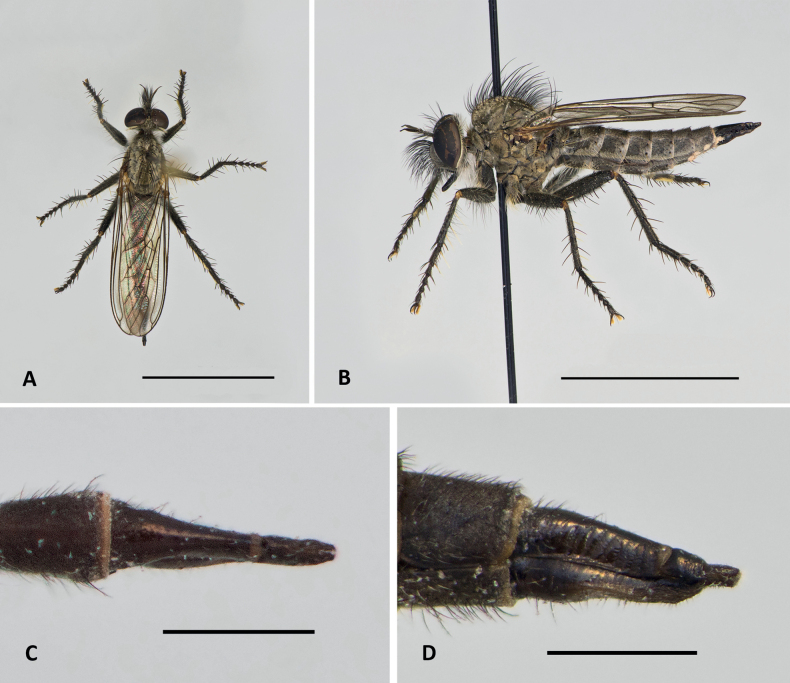
*Conosiphonianus* Álvarez Fidalgo & van den Broek, sp. nov. Female paratype (MNCN_Ent 344924) **A** habitus in dorsal view **B** habitus in lateral view Female paratype from Aguadulce in PAFC**C** Ovipositor, dorsal view **D** ovipositor, lateral view. Scale bars: 5 mm (**A, B**); 1 mm (**C, D**). Photographs: PÁF.

**Figure 5.**
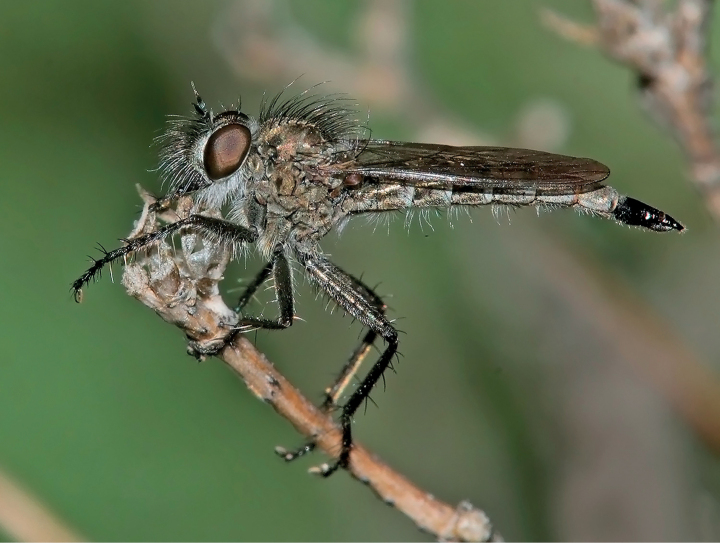
*Conosiphonianus* Álvarez Fidalgo & van den Broek, sp. nov. Female in its natural habitat, Aguadulce (Roquetas de Mar), Almería, Spain, 7-XII-2015. Photograph: PÁF.

**Figure 6.**
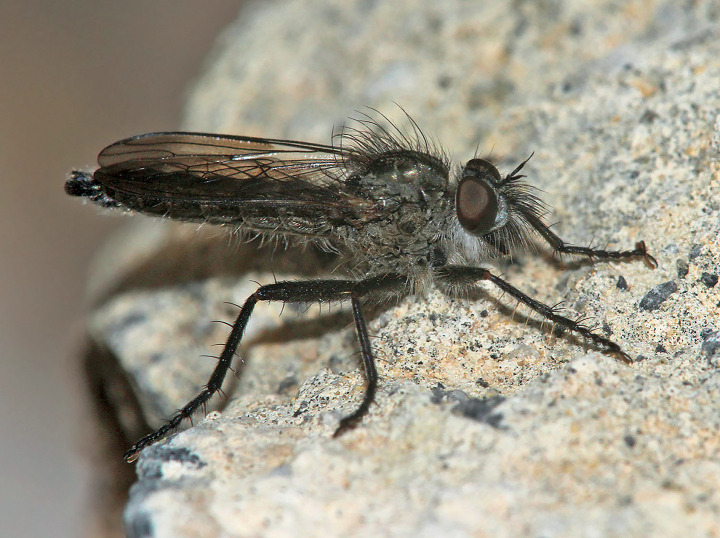
*Conosiphonianus* Álvarez Fidalgo & van den Broek, sp. nov. Male (dark-winged form) in its natural habitat, Aguadulce (Roquetas de Mar), Almería, Spain, 7-XII-2015. Photograph: PÁF.

Figs 1 , 2 , 3 , 4 , 5 , 6

#### Examined material.

**Type material. *Holotype*** Almería, Spain • 1 ♂; Cabo de Gata; 36°49.3'N, 2°15.4'W, 20 m a.s.l.; 02.I.2022; F. Rodríguez leg.; MNCN_Ent 344922; pinned. ***Paratypes*** Almería, Spain • 2 ♂; same collection data as for holotype • 5 ♀♀; same collection data as for holotype; pinned. The holotype, 1 ♂ paratype and 4 ♀♀, are stored in MNCN (MNCN_Ent 344923-27), the remainder in PAFC. • 1 ♂; Aguadulce (Roquetas de Mar); 36°49.7'N, 2°35.2'W, 170 m a.s.l.; 03.XII.2015; F. Rodríguez leg.; MNCN_Ent 361026; pinned and dissected, genitalia stored in phial. • 1 ♂ + 1 ♀; same collection data; 25.XII.2015; RVDBC; pinned. • 3 ♂ + 3 ♀; same collection data; 01.XII.2016; RVDBC; pinned. • 3 ♂; same collection data; 11.XII.2016; RMNH; specimens in ethanol. • 2 ♂ + 2 ♀; same collection data; 21.IX.2017; 1 ♂ + 1 ♀ at ZMHB, 1 ♂ + 1 ♀ at USNM; pinned. • 2 ♂ + 1 ♀; same collection data; 07.XII.2017; PAFC; pinned. • 1 ♀; same collection data; 12.I.2019; PAFC; pinned. • 2 ♂; Almerimar (El Ejido); 36°41.7'N, 2°45.9'W, 40 m a.s.l.; 14.XII.2017; F. Rodríguez leg.; MNCN (MNCN_Ent 344928-29); pinned. • 2 ♂; Cabo de Gata; 36°49.6'N, 2°13.6'W, 30 m a.s.l.; 29.XI.2015; F. Rodríguez leg.; RMNH; stored in alcohol. • 2 ♂ + 2 ♀; same collection data and date; RVDBC; pinned. • 1 ♂; El Parador (Vícar); 36°49.2'N, 2°36.1'W, 115 m a.s.l.; 22.XI.2015; F. Rodríguez leg.; RVDBC; pinned. • 1 ♂ + 2 ♀; same collection data; 05.I.2019; F. Rodríguez leg.; PAFC; pinned. • 3 ♂ + 3 ♀; same collection data; 05.I.2020; F. Rodríguez leg.; RMNH; in ethanol. • 1 ♂; Rambla Morales, Cabo de Gata; 36°49.6'N, 2°13.6'W, 30 m a.s.l.; 08.XII.2015; P. Álvarez leg.; RMNH; in ethanol. • 1 ♂ + 1 ♀; Rambla del Águila, Santa María del Águila (El Ejido); 36°49.0'N, 2°46.2'W, 380 m a.s.l.; 10.XII.2015; P. Álvarez leg.; PAFC; pinned. • 1 ♂; Retamar, Cabo de Gata; 36°49.6'N, 2°13.6'W, 30 m a.s.l.; 08.XII.2015; P. Álvarez leg.; RMNH; in ethanol. • 1 ♀; Sorbas; 37°08.5'N, 2°02.6'W, 365 m a.s.l.; 01.I.2019; F. Rodríguez leg.; MNCN_Ent 344930; pinned. • 1 ♂; Tabernas; 36°49.2'N, 2°36.1'W, 115 m a.s.l.; 19.XI.2015; F. Rodríguez leg.; MNCN_Ent 361025; pinned and dissected, genitalia stored in phial.

#### Diagnosis.

Head proportionally large; facial protuberance strong and prominent, occupying most of the face, its upper margin very well developed, more than in any other species of *Conosiphon*; ventral macrosetae of fore femora absent, only long and thin setae present; macrosetae of mystax very dense and at least as long as the antennae, or longer; legs entirely dark, only basal part of the tibiae narrowly pale coloured; acrostichal macrosetae long and abundant. Hypopygium with a small and pointy epandrial lobe on the dorsal inner side, just before the apex. Hypandrium with a blunt projection on hind margin.

#### Description.

**Male** (Figs 1–3, 6). Length of the body: 9 mm, length of the wing: 6.5 mm. ***Head*.** Frons, facial protuberance, genae and occiput, covered with pale grey tomentum. Ocellar tubercle rather high, reaching eye level, and bearing several long and erect black piliform macrosetae. Frons with some much shorter mainly black true setae. Facial protuberance strong and prominent, occupying most of the face, its upper margin well developed; distance of the upper margin to antennal socket slightly shorter than the length of the pedicel. Mystax dense and black, consisting of black piliform macrosetae, as long as or even longer than the antennae, in the lower part mixed with very few pale ones. Eyes dark brown. Face narrow, its width somewhat less than half one eye. Short macrosetae placed on top of occiput black, some shorter and white ones on both sides. All setae on the back of the head white, longer on top, shorter behind, the lower occipital macrosetae and setae white and very long. Proboscis and palpi black, both with some white setae on tip.

Antenna black, covered with grey tomentum. Ratio scape: pedicel: flagellum, 2:1:5. Scape ~ 5× longer than broad, cylindrical in lateral view and bearing some stiff white seta-like macrosetae dorsally; more and longer mainly black ones ventrally. Pedicel much shorter (~ 1/2 the length of the scape), conical (narrower on the base and broader apically) in lateral view, bearing only short and stiff black macrosetae on the apical area, both dorsally and ventrally. Flagellum bare and nearly as long as the two basal antennal segments together. Postpedicel ~ 3× the length of the pedicel. Style three-segmented (1:4:1), bare and nearly half as long as the postpedicel, last segment very narrow.

***Thorax*.** Antepronotal macrosetae pale, well developed and mixed with numerous pale setae. Postpronotal lobe with some longish white setae. Scutum, scutellum, and pleurae black, covered with dense brownish grey tomentum. Dusting less dense along the median line of the scutum, giving the appearance of having a dark middle band along the mesonotum. Mesonotal setae very thin, scattered, and black, some pale ones hardly noticeable. On the mesonotal line, five pairs of presutural dorsocentrals, and six or seven pairs of post-sutural ones. The post-sutural macrosetae are thinner in the prescutellar area, here similar to acrostical macrosetae. The acrostical macrosetae are black, slightly shorter and stouter than dorsocentral ones and more abundant than mesonotal setae. All macrosetae located over the mesonotal middle line from the pronotal area to the prescutellar area. Scutellar surface bearing several long and soft white setae; scutellar margin with four stout and very long black macrosetae (and a fifth central thick seta that might be considered a macroseta). Notopleural, supraalar, and postalar macrosetae long and black. Anepisternum and katepisternum almost bare, with only some isolated thin pale setae, and a few dark macrosetae-like setae on dorsal area of anepisternum. Anepimeron with a set of seven or eight pale dorsal macrosetae and katatergite with a row of seven or eight long pale macrosetae. Halteres brown.

***Legs*.** Coxae heavily grey tomentose, with several long macrosetae and setae. Legs shiny black and thin, covered with short pale setae. Only the area of connection of femora and tibiae pale, with apex of femora and basal part of the tibiae both narrowly yellowish. No ventral macrosetae on fore femur, but long, thin, black and white setae are present. Macrosetae on femora, tibiae, and tarsi stout, predominantly pale on fore and mid legs, and predominantly black on the hind legs. Claws black. Pulvilli brownish yellow.

***Wing*.** Hyaline, slightly darkened in the apical area. Costa with black fine setae. Veins dark brown, only pale brown on the basal area.

***Abdomen*.** Abdomen black, covered with dense brownish grey tomentum, like the mesonotum; tomentum less dense on the dorsal area of each tergite, giving an appearance of having dark patches. Tergal setae whitish, except on the dorsal area of the tergites, where they are longer, thicker, and black. Tergite I with dorsal setae particularly longer, pale, and more erect on the basal part, black and more adpressed on the marginal area. Tergites II and III with shorter black setae dorsally and more adpressed than those on tergite I. Dorsal black setae even shorter and more adpressed on tergites IV-VIII. Tergites I-VI with two or three thick, very long, pale marginal macrosetae laterally. Sternite I with very long, erect and dense whitish filiform setae, sternite II bare, the remaining sternites with long, thin, erect white setae, but shorter than those on sternite I. Sternites IV-VII with a pair of long true macrosetae, not as strong as those on terga, directed outwards and placed near the margin of the sternites; on the remaining sternites, macrosetae thinner and hardly differentiated from the true setae.

***Terminalia*** (Fig. 3). Hypopygium black, covered with stiff white setae. Phallus somewhat tubular, tapering at the tip (Fig. 3A, B). Epandrium with a small, curved, and pointed lobe, located on the dorsal inner side, just before the apex (Fig. 3C), simple in lateral view (Fig. 3D). Gonostylus long, rounded at the tip, with a small depression in the middle (Fig. 3E). Hypandrium with a blunt projection on hind margin, bearing thin white setae; anterior margin distinctly concave (Fig. 3F).

**Female (Figs 4, 5).** Very similar to male, slightly larger, only differs in that sternal macrosetae that are less developed and frequently black. The ovipositor is illustrated in Fig. 4C, D.

#### Variation.

The main variation involves the colouration of the wings of the males (Figs 1, 2, 6). It is remarkable that two colour forms coexist in all locations, one with hyaline wings (Fig. 2C), which seem to be slightly commoner, and the other with darkened wings (Fig. 2D), only hyaline on 1/3 of the basal area. Intermediate forms are rarely found, but they do exist. All females have hyaline wings. No relevant differences have been found in the genitalia of the males of both forms, nor do they differ in COI barcodes (see Fig. 11 and adjoining molecular analyses).

The number of dorsocentral macrosetae vary greatly, both in male and female, from 4–6 pairs of presutural, and six or seven pairs of postsutural ones, some thicker, some thinner, but always thinner on the prescutellar area, where they are approximately the same length or slightly shorter than the dorsocentrals (as mentioned in the description). It is remarkable that the number and position of the dorsocentral macrosetae are not even symmetrical within one single specimen.

The number of marginal scutellar macrosetae is very variable too in both sexes (2–6, sometimes 3 or 5, usually 4), and frequently some of them are slightly thinner and it is doubtful whether they can be considered macrosetae or not. Rarely one or two can be pale instead of black. Also, leg macrosetae are variable in colouration and in some specimens, occasionally the number of pale macrosetae on hind legs is higher than usual. Legs are entirely black or at most the knees are narrowly pale. There is no correlation between the dark-winged form and the darker colouration of the legs, nor for the clear-winged form and the lighter knees.

#### Size variation.

Length of the body: 8.5–9.5 mm, length of the wing: 6.0–6.5 mm.

#### Other material studied.

**Figure 7.**
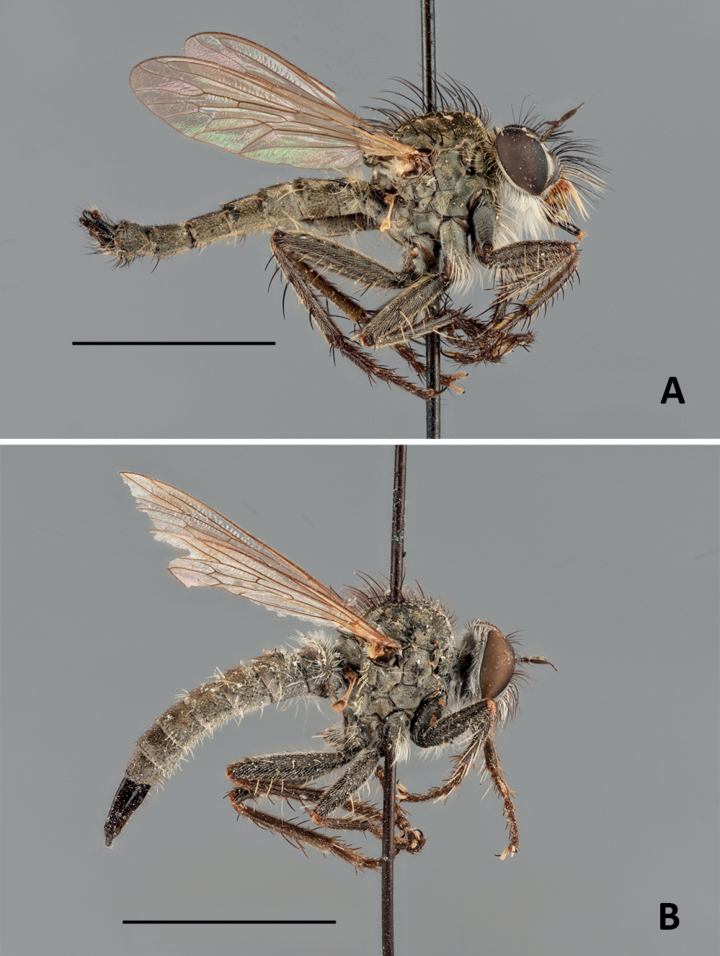
**A** holotype of *Conosiphonpauper* (Becker, 1907), male habitus in lateral view **B** syntype of *Conosiphonsimilis* Becker, 1923, female habitus in lateral view. Scale bars: 5 mm. Photographs: E. Wolff, courtesy of J. Pohl, ZMHB.

Type specimen of *Conosiphonpauper* (Becker, 1907). ***Holotype*** Algeria • 1 ♂ (Fig. 7A); Algier IV, 52370; Dysm. pauper Beck det. Becker; Holotypus [red label]. Type specimen of *Conosiphonsimilis* Becker, 1923. ***Syntype*** Algeria • 1 ♀ (Fig. 7B); El-Kantara, 52631; similis Beck; Typus [red label]. Both specimens studied through high resolution images provided by courtesy of Jenny Pohl, ZMHB.

#### Etymology.

The new species is named after the Roman deity Janus for two reasons: on the one hand, because Janus gives the name to the month of January, the peculiar flight period (mainly December and January) of this robber fly, and on the other hand, because Janus is represented showing two faces, expressed in *C.ianus* by the presence of two colour forms (clear-winged and dark-winged). The name *ianus* should be treated as a noun in apposition (ICZN 1999).

#### Habitat, biology, and ecology.

**Figure 8.**
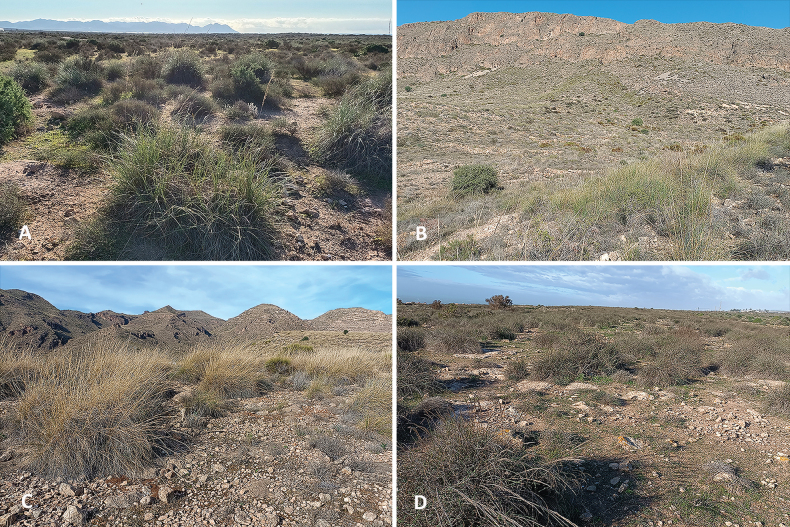
Four locations showing the habitat of *Conosiphonianus* Álvarez Fidalgo & van den Broek, sp. nov. in the province of Almería, Spain **A** type location in Cabo de Gata **B** aguadulce **C** vícar **D** almerimar. Photographs: F. Rodríguez Luque.

The first evidence of the presence of this species dates back to the winter of 2009 (Fig. 1) when F. Rodríguez Luque photographed a peculiar looking male Asilinae in Roquetas de Mar. The typical habitat where he found the same species in later years consists of open ground with scarce bushy vegetation but with extensive esparto grass (*Stipatenacissima*). This habitat is frequently found near the coast but can also be found in the interior, in ramblas and desertic areas with gypsiferous soil (Fig. 8).

Copulas were frequently observed, taking place always on the ground. Also, as occurs in other species of Asilidae, cannibalism was sometimes observed. Due to the limited number of hunting observations, little is known about their main prey. However, observations preserved in photographs exist of *C.ianus* feeding on Coleoptera, Diptera (*Bibio* sp. (presumably *Bibiogineri* Gil Collado, 1932), Coenosinae, *Musca* sp., and Sarcophagidae), Heteroptera, and Hymenoptera.

#### Distribution and phenology.

**Figure 9.**
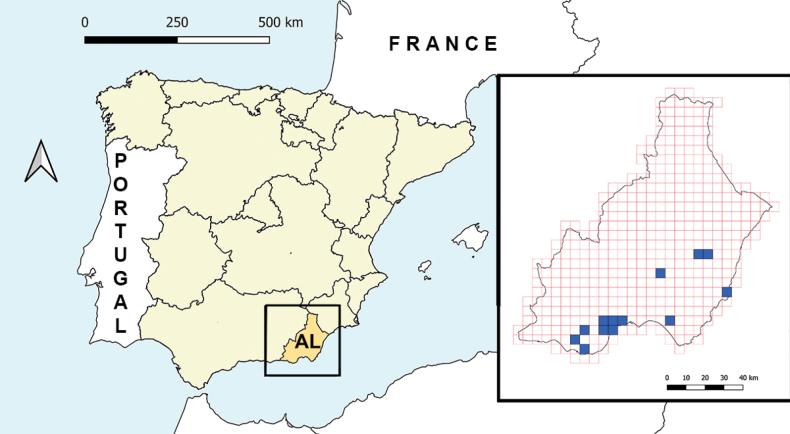
Distribution map of *Conosiphonianus* Álvarez Fidalgo & van den Broek, sp. nov. in Spain, created from all available data, both collected specimens and published photographs. Map: M. Álvarez Fidalgo.

Up to this date, *C.ianus* Álvarez Fidalgo & van den Broek sp. nov. is only known from the province of Almería, southeastern Spain. It seems to be present in several coastal areas of the south of the province, being much more local in the east coast and in the semi-desertic interior of the province In order to provide a clearer view of the known distribution of this species, all available records were gathered and then located in a 5 × 5 km grid map of the province of Almería (Fig. 9). Records come from two sources, collected specimens and photographs taken in the wild uploaded to the online database BiodiversidadVirtual.org (2023). The locations of all the pinned specimens studied were already shown under the chapter ‘Type material’. Suppl. material 1 provides information related to all material identified from photographs at BiodiversidadVirtual.org (2023). Only photographs where the specimens can be positively identified were used.

**Figure 10.**
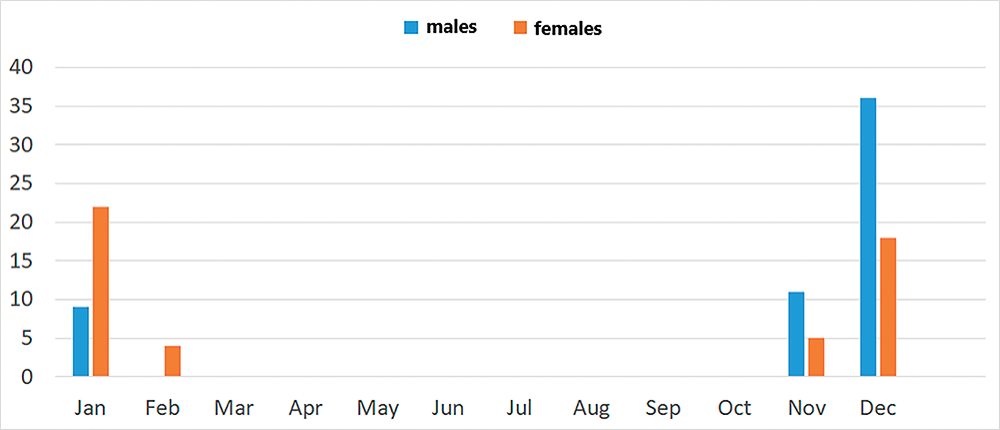
Graphic showing the flight period of *Conosiphonianus* Álvarez Fidalgo & van den Broek, sp. nov., based on the 105 records gathered from collections and photographs. Graph: M. Álvarez Fidalgo.

As can be seen from all the available records, this is a winter species. The earliest date it was found was 8 November and the latest 6 February. However, usually the first adults start to be seen in the third week of November. Normally, the peak of abundance occurs in December and the species is found still in good numbers till middle January. Numbers decline rapidly in the third week of January and the species is rarely seen during the first week of February. Fig. 10 shows the phenology of the species (based on the available records) in a graphic.

#### Molecular analysis.

**Figure 11.**
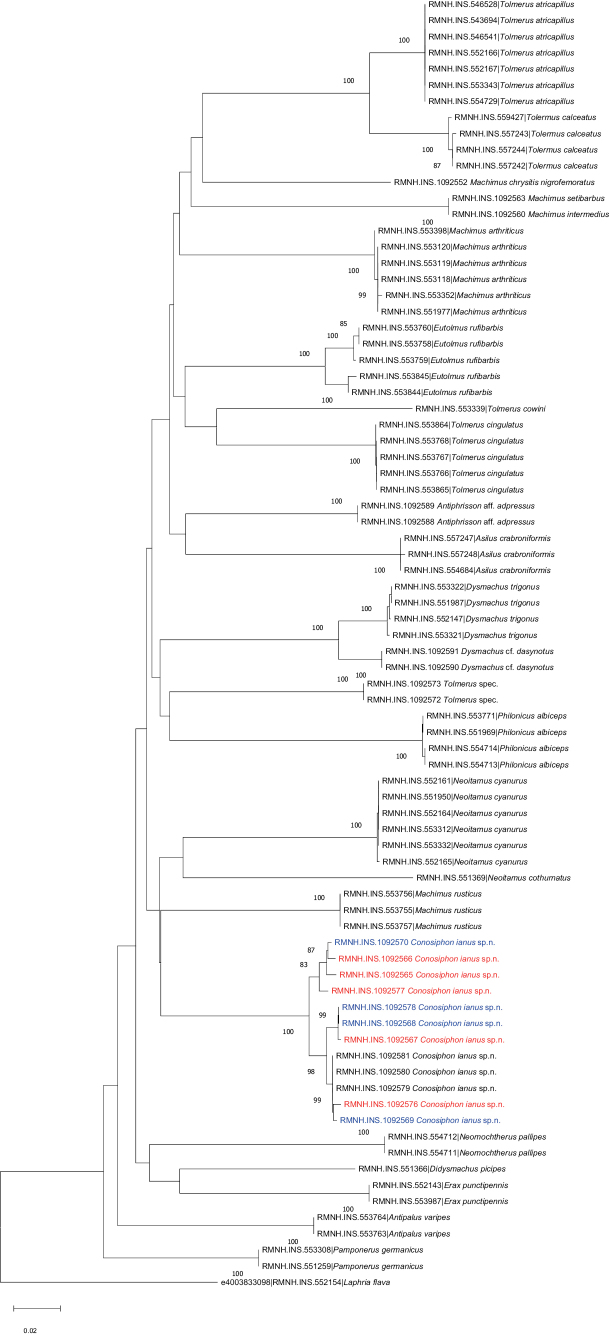
Neighbour-Joining tree for *Conosiphonianus* Álvarez Fidalgo & van den Broek, sp. nov., and related genera of the subfamily Asilinae. *Laphriaflava* (Linnaeus, 1761) is used as an outgroup. Red indicates dark-winged males and blue indicates clear-winged males of *C.ianus*.

Due to presence of two distinct morphological forms within the range of *C.ianus* sp. nov. (e.g., the colouration of the wings in the male), we analysed 12 specimens, including nine males: four clear-winged forms and five dark-winged forms. All specimens clearly group together in one clade, separate from other taxa in all analyses executed: Neighbour-Joining, Minimum Evolution and Maximum Likelihood. The results are presented in a Neighbour-Joining tree (Fig. 11), full details of the specimens of *C.ianus* sp. nov. used for DNA barcoding are provided as a supplementary .xlsx file (Suppl. material 2). There is considerable variation among the barcodes of *C.ianus* sp. nov. with three groups consistently resolved with bootstrap values of 100 and 98 respectively and an average K2P distance of 1.7% (0.9–2.3). The average distance among all Asilinae species included in the analyses is 19.8% (4.0–27.7). The three groups within *C.ianus* sp. nov. do not comply with the differences in wing colouration among the males, as indicated in Fig. 11: clear-winged (blue), dark-winged (red). Therefore, although there is considerable variation among the barcodes of *C.ianus* sp. nov., we consider it to be a single variable species with two distinct colour forms in the males. Future analyses, including DNA barcodes of other congeneric species, should resolve whether or not *C.ianus* sp. nov. is indeed one single species or perhaps a complex of species.

**Figure 12.**
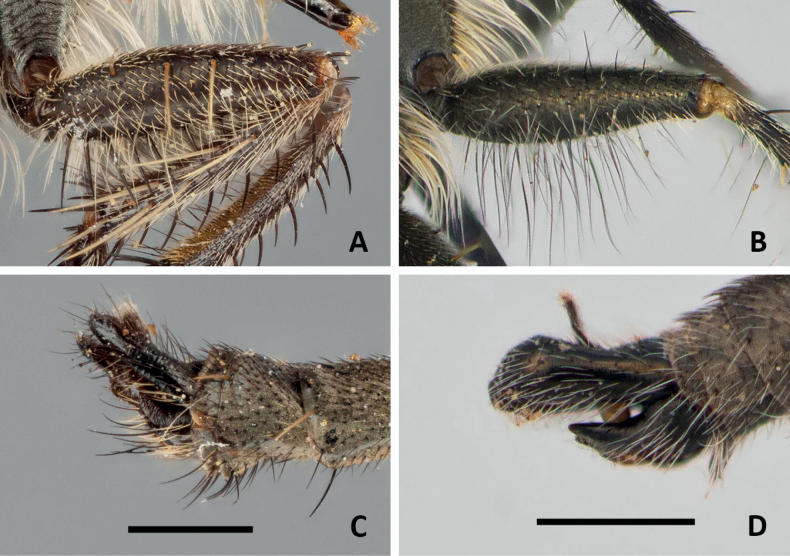
Holotype (male) of *Conosiphonpauper* (Becker, 1907) **A** fore femora, showing macrosetae present on ventral side **C** terminalia in lateral view. Photos: E. Wolff, courtesy of J. Pohl, ZMHB. Paratype (male) of *Conosiphonianus* Álvarez Fidalgo & van den Broek, sp. nov. **B** fore femora, showing filiform setae present on ventral side **D** terminalia in lateral view. Scale bars: 1 mm. Photographs: PÁF.

**Figure 13.**
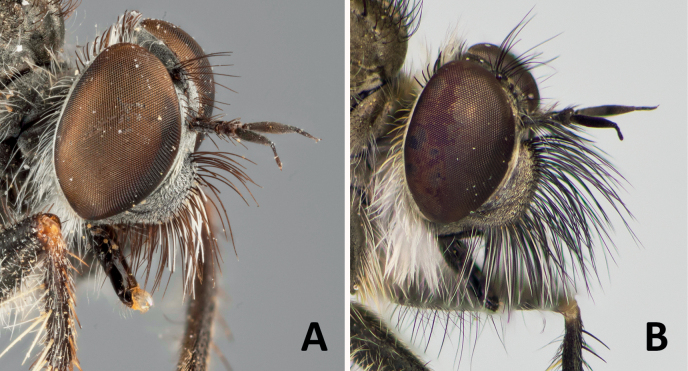
Syntype (female) of *Conosiphonsimilis* Becker, 1923 **A** head in frontolateral view. Photo: E. Wolff, courtesy of J. Pohl, ZMHB. Paratype (female) of *Conosiphonianus* Álvarez Fidalgo & van den Broek, sp. nov. **B** head in frontolateral view. Photograph: PÁF.

### ﻿Identification key to the known species of *Conosiphon*

This key is provided for all species tentatively placed in the genus *Conosiphon*.

**Table:** 

1	Macrosetae present on ventral side of front femora (Fig. 12A). Hypandrium without projection on hind margin or, if present, it is very poorly developed (Fig. 12C)	**2**
–	Macrosetae absent on ventral side of front femora, only filiform setae present (Fig. 12B). Hypandrium with a clearly developed blunt projection on hind margin [beware the unknown male of *C.similis*] (Fig. 12D)	**3**
2	Medium sized species, 13–14 mm. On ventral side of fore femora a double row of macrosetae, yellow and black in male, all black in female. Style of antennae ~ 1/2 as long as postpedicel. Known distribution: Algeria. Flight period: unknown	***Conosiphonalter* Becker**
–	Smaller species, 10–11 mm. On ventral side of fore femur a single row of strong black macrosetae (Fig. 12A). Style of antennae clearly longer than 1/2 the length of the postpedicel. Terminalia (Fig. 12C). Known distribution: Morocco?, Algeria, and Tunisia. Flight period: collected in spring	***Conosiphonpauper* Becker**
3	Facial protuberance well developed but sloped in the upper margin; distance between dorsal ridge and antennal socket approximately equal to the length of the scape (Fig. 13A). Mystax not very dense and macrosetae shorter than the length of the antennae. Acrostichal macrosetae seta-like and much shorter than dorsocentral macrosetae. Male unknown. Known distribution: Algeria and Tunisia. Flight period: unknown	***Conosiphonsimilis* Becker**
–	Facial protuberance very large in both sexes, upper ledge very well developed; distance between dorsal ridge and antennal socket clearly shorter than the length of the scape (Fig. 13B). Mystax very dense and macrosetae as long as or longer than the antennae. Acrostichal macrosetae almost as long and stout as the dorsocentral macrosetae. Terminalia (Fig. 12D). Known distribution: Almería, Spain. Flight period: collected from November to February (winter)	***Conosiphonianus* Álvarez Fidalgo & van den Broek**

## ﻿Discussion

As previously commented, we treat the new species as *Conosiphon* after Hull (1962). However, this genus may not yet be well defined. As has been made clear from the taxonomy section under the genus *Conosiphon*, the generic limitations have been subject to debate for a long time. Here we treat all species with a prominent facial protuberance, the mesonotum entirely covered with dorsocentral macrosetae, and the female cerci not partly incorporated in the ovipositor in tergite IX, but sticking out at the apex as belonging to the genus *Conosiphon*. The species assigned to the genus *Conosiphon* at some point in history, discussed above and now considered to belong to other genera, failed to a greater or lesser extent to fulfil these three requirements as deduced from what can be read in the literature. Nevertheless, more poorly studied species might also prove to belong to this genus. This, however, can only be resolved through a thorough revision including examination of the types of all species assigned to the genus at some point in time, as well as species belonging to closely related genera, which is outside the scope of this paper. Here we merely describe a new species, compare it to the type material of the available species that fit the concept of the genus *Conosiphon* and provide a key to all those species. Future research should include morphology as well as DNA barcoding of all related species in order to establish the true identity of the genus *Conosiphon* and its species.

The most important fact is that a new species of robber fly of an unknown genus in Europe has been found in Almería, Spain. For the first time a species of *Conosiphon* has been described in detail, illustrated, and its ecological information provided. Regarding the problem of making sure *C.ianus* is truly a valid species but not being able to examine the types directly, high resolution images turned out to be a very useful tool. The descriptions by Becker were concise enough but some important details were not mentioned, for example, the shape of the hypandrium of the males. However, these doubts were solved thanks to the photographs. The macrosetae of the ventral side of the fore femora are as strong as they should be (Fig. 12A), very different from the filiform setae of *C.ianus* (Fig. 12B). The shape of the epandrium in lateral view is much thinner and it is narrower at the tip in *C.pauper* (Fig. 12C) than in *C.ianus*, which has a much more rectangular shaped epandrium in lateral view (Figs 3D, 12D). Moreover, there is no projection in the hind margin of the hypandrium of *C.pauper* (Fig. 12A) whereas it is very obvious in *C.ianus* (Figs 3F, 12D). Probably Becker did not mention this character in the description of *C.pauper* because there was nothing remarkable about it. Additionally, it is important to mention the shape of the facial protuberance, which is remarkably developed in the upper ledge in *C.ianus* (Fig. 2B) and sloped in *C.pauper* (Fig. 7A).

Dealing with *C.similis* was much more difficult as the male is unknown and only females could be compared. Despite difficulties, there are clear differences in the shape of the facial protuberance, which is, as in the male, very well developed in the female of *C.ianus*. In the female of *C.similis*, as in the male of *C.pauper*, it slopes from the upper side. The mystax of the type specimen studied of *C.similis* is somewhat damaged (Fig. 13A) but still many macrosetae are complete and it can be seen in the image that they are shorter than the antennae and not abundant. Only a few sockets are present which indicates that not many macrosetae are really missing. *Conosiphonianus*, however, has longer and more abundant macrosetae on mystax (Fig. 13B). Additionally, Becker (1923) mentions that mesonotal macrosetae are not very long. Most are broken in the type specimen studied but in Fig. 7B it can be seen that the prescutal macrosetae are in good condition and they are much shorter than the ones in *C.ianus* (Fig. 4B).

Unfortunately, the type material of *C.alter* seems to be lost as the specimens used by Becker (1923) to describe this taxon are not present in the collection Villeneuve in Brussels. Therefore, at least for the moment, it is not possible to compare *C.ianus* with it. Nevertheless, the types might be found somewhere else in the future. In spite of this inconvenience, from Becker (1923) it is clearly deduced that he studied at least one male and one female, which were rather large specimens (13–14 mm) compared with the much smaller *C.similis* (10 mm) and *C.ianus* (8.5–9.5 mm). Moreover, the double row of macrosetae of the ventral side of the fore femora are present in all specimens, otherwise he should have mentioned this anomaly somewhere, which shows that this feature is at least rather stable. No macrosetae on ventral side of the fore femora were observed in any specimen of *C.ianus*. And as can be seen in Fig. 12A of *C.pauper*, the idea of macrosetae by Becker is not subjective, but real. This difference is enough to deduce that we are dealing with two different species. Moreover, the fact that also in this species Becker does not mention the existence of a projection in the hind margin of the hypandrium of *C.alter* makes us assume that it is absent and therefore another important difference helps to separate both species.

It is also important to comment about the status of the new species. Between 2009 and 2014 it was observed regularly but never in high numbers. However, in 2014 the species seemed to go through a burst in population size, and it was common in several areas. It was found in good numbers until 2018–2019 but seems to be severely declining in recent years. Its decline in Roquetas de Mar and El Ejido might well be related to habitat destruction and human activities. However, these factors did not happen yet in other areas of the province where it was previously found. In our opinion, this species might be affected by the lack of rain as Almería is going through a severe drought in recent years. Effectively, the years when the species was common did concur with somewhat rainy November months. A few weeks later, the imagoes started to be observed, usually the first week of December. Nevertheless, November in 2019–2022 has been much drier than usual, particularly 2021–2022, when imagoes were hardly seen.

Therefore, the province of Almería, as part of the Mediterranean Basin, is an exceptional hotspot of biological diversity that still is providing unexpected new species to science in spite of the current biodiversity and climate crisis. But even species that were common not long ago are being severely affected by human pressure and climate change. *C.ianus* sp. nov. seems to be a good example of this.

## Supplementary Material

XML Treatment for Conosiphon

XML Treatment for Conosiphon ianus

## ﻿Additional information

### Author ORCIDs

Reinoud van den Broek https://orcid.org/0009-0000-0970-9356

Piluca Álvarez Fidalgo https://orcid.org/0000-0002-5330-3078

John Smit https://orcid.org/0000-0002-1568-5183

### Data availability

All of the data that support the findings of this study are available in the main text or Supplementary Information.

## Supplementary materials

Supplementary material 1 Data compilation of live specimens of *Conosiphonianus* Álvarez Fidalgo & van den Broek, sp. nov. Data type xlsx Explanation note Identified from photographs available in the public website BiodiversidadVirtual.org. The images were taken by F. Rodríguez Luque. https://binary.pensoft.net/file/915213This dataset is made available under the Open Database License (http://opendatacommons.org/licenses/odbl/1.0/). The Open Database License (ODbL) is a license agreement intended to allow users to freely share, modify, and use this Dataset while maintaining this same freedom for others, provided that the original source and author(s) are credited.Author: Piluca Álvarez Fidalgo

Supplementary material 2 Full details of the specimens of *Conosiphonianus* Álvarez Fidalgo & van den Broek, sp. nov. used for DNA barcoding Data type xlsx https://binary.pensoft.net/file/915214This dataset is made available under the Open Database License (http://opendatacommons.org/licenses/odbl/1.0/). The Open Database License (ODbL) is a license agreement intended to allow users to freely share, modify, and use this Dataset while maintaining this same freedom for others, provided that the original source and author(s) are credited.Author: John Smit

## References

[B1] Anichtchenko A (2005) Especies nuevas y poco conocidas de *Philorhizus* Hope, 1838 (Coleoptera, Carabidae) de España. Boletín Sociedad Andaluza de Entomología 12: 46–50.

[B2] Becker T (1907) Die Ergebnisse meiner dipterologischen Frühjahrsreise nach Algier und Tunis. 1906. Zeitschrift für Systematische Hymenopterologie und Dipterologie 7: 33–61. [part] 10.5962/bhl.title.9280

[B3] Becker T (1923) Revision der Löw’schen Diptera Asilica in Linnaea Entomologica 1848–49. Wien, F. Wagner, 91 pp.

[B4] Biodiversidad Virtual (2023) Online database. http://www.biodiversidadvirtual.org [Accessed 06 Jan 2023]

[B5] van den Broek R Álvarez Fidalgo P (2018) *Stenopogondenudatus* Loew, 1856 confirmed as a valid species separate from *Stenopogongruenbergi* Becker, 1911, and description of a new species of *Stenopogon* Loew, 1847 from Spain (Diptera: Asilidae). Boletín de la Sociedad Entomológica Aragonesa (S.E.A.) 63: 187–197.

[B6] Brooks TM Mittermeier RA Mittermeier CG da Fonseca GA Rylands AB Konstant WR Flick P Pilgrim J Oldfield S Magin G Hilton-Taylor C (2002) Habitat loss and extinction in the hotspots of biodiversity. Conservation Biology 16(4): 909–923. 10.1046/j.1523-1739.2002.00530.x

[B7] Carles-Tolrá M (2020) *Iberoteluscinectatus* sp. n.: a new therevid species from Spain, with a key to the known species of the genus (Diptera: Therevidae). Boletín de la Sociedad Entomológica Aragonesa (S.E. A) 66: 43–50.

[B8] Castillo Requena JM (1989) El clima de Andalucía: Clasificación y Análisis Regional con los Tipos de Tiempo. Instituto de Estudios Almerienses, Almería, 500 pp.

[B9] Castro Tovar A (2014) Una nueva especie de *Blaps* Fabricius, 1775 del sureste de España (Coleoptera, Tenebrionidae). Arquivos Entomolóxicos 12: 237–243.

[B10] Cueto M Blanca G Salazar C Cabezudo B (2014) Diversity and ecological characteristics of the vascular flora in the Western Mediterranean (Eastern Andalusia, Spain). Acta Botanica Malacitana 39(1): 81–97. 10.24310/abm.v39i1.2574

[B11] Cumming JM Monty Wood D (2017) Adult morphology and terminology [chapter 3]. In: Kirk-Spriggs AH Sinclair BJ (Eds) Manual of Afrotropical Diptera (Vol.1). Introductory chapters and keys to Diptera families. Suricata 4. South African National Biodiversity Institute, Pretoria, 89–133.

[B12] Engel EO (1930) Asilidae: Diptera. In: Lindner E (Ed.) Die Fliegen der Paläarktischen Region. Stuttgart, Schweizerbart’sche Verlagsbuchhandlung, 491 pp.

[B13] Evenhuis NL Pape A (2023) Systema Dipterorum. The BioSystematic Database of World Diptera. Version 4.0. [Online database]. http://www.diptera.org [Accessed 05 Feb 2023]

[B14] Folmer O Black M Hoeh W Lutz R Vrijenhoek R (1994) DNA primers for amplification of mitochondrial cytochrome c oxidase subunit I from diverse metazoan invertebrates. Molecular Marine Biology and Biotechnology 3: 294–299. 7881515

[B15] Hebert PDN Cywinska A Ball SL deWaard JR (2003) Biological identifications through DNA barcodes. Proceedings of the Royal Society of London, Series B, Biological Sciences 270(1512): 313–321. 10.1098/rspb.2002.2218 PMC1691236 12614582

[B16] Hebert PDN Penton EH Burns JM Janzen DH Hallwachs W (2004) Ten species in one: DNA barcoding reveals cryptic species in the neotropical skipper butterfly *Astraptesfulgerator*. Proceedings of the National Academy of Sciences of the United States of America 101(41): 14812–14817. 10.1073/pnas.0406166101 15465915 PMC522015

[B17] Hradský M Bosák J (2006) Distribution of some species of robber-flies (*Diptera*, *Asilidae*) in the Canary Islands, and description of three new species of genus *Tolmerus* Loew, 1849. Časopis Slezského zemského muzea Opava (A) 55: 135–145.

[B18] Hull FM (1962) Robber flies of the world. The genera of the family Asilidae. Bulletin – United States National Museum 224: 1–907. 10.5479/si.03629236.224

[B19] ICZN (1999) International Code of Zoological Nomenclature (4^th^ edn.). International Trust for Zoological Nomenclature, London, 306 pp. https://www.iczn.org/the-code/the-code-online/

[B20] Izquierdo I Martin JL Zurita N Arechavaleta M (2001) Lista de especies silvestres de Canarias (hongos, plantas y animales terrestres). Consejería de Politica Territorial y Medio Ambiente, Gobierno de Canarias, 437 pp.

[B21] Lehr PA (1967) Ecological and morphological study of Robber Flies of the Tribe Asilini (Diptera, Asilidae) with descriptions of new genera and species from Kazakhstan and Soviet central Asia. Entomological Review 46(2): 232–241. [Translated from Russian]

[B22] Lehr PA (1988) Family Asilidae. In: Soós A, Papp L (Eds) Catalogue of Palaearctic Diptera (Vol. 5). Athericidae – Asilidae. Akadémiai Kiadó, Budapest, 446 pp.

[B23] Lehr PA (1992) „Small“ genera of Robber Flies of the Subfamily Asilinae (Diptera: Asilidae). 1. Taxonomy and Ecology. Зоологический журнал 71: 91–105. [In Russian]

[B24] López-López P Maiorano L Falcucci A Barba E Boitani L (2011) Hotspots of species richness, threat and endemism for terrestrial vertebrates in SW Europe. Acta Oecologica 37(5): 399–412. 10.1016/j.actao.2011.05.004

[B25] Myers N Mittermeier RA Mittermeier CG da Fonseca GAB Kent J (2008) Biodiversity hotspots for conservation priorities. Nature 403(6772): 853–858. 10.1038/35002501 10706275

[B26] Pagola-Carte S (2017) *Adelphocorisfalukei* n. sp. from Almería, southeastern Iberian Peninsula (Hemiptera: Heteroptera: Miridae). Heteropterus Revista de Entomología 17(2): 113–129.

[B27] Pollet M Andrade R Gonçalves A Álvarez Fidalgo P Camaño Portela JL Belin F Mortelmans J Stark A (2022) Discovery of a Lineage of Soil-Dwelling *Medetera* Species with Multi-Coloured Eyes in Southern Europe (Diptera: Dolichopodidae). Insects 13(11): e1012. 10.3390/insects13111012 PMC9699082 36354836

[B28] Wood TJ Ghisbain G Michez D Praz C (2021) Revisions to the faunas of *Andrena* of the Iberian Peninsula and Morocco with the descriptions of four new species (Hymenoptera: Andrenidae). European Journal of Taxonomy 758: 147–193. 10.5852/ejt.2021.758.1431

